# A case report of sanguisis streptococcal sepsis associated with the orthodontic appliance in a young male with aortic valve disease

**DOI:** 10.1515/med-2026-1463

**Published:** 2026-06-15

**Authors:** Bassem Al Hariri, Yussuf Abdi Hassan, Abdelkarim Mohamed, Muad Abdi Hassan

**Affiliations:** Department of Medicine, Hamad Medical Corporation, Doha, Qatar; Weill Cornell Medicine – Qatar, Doha, Qatar; General Practice Department, Al Jameel Medical Center, Doha, Qatar; Medical Education Department, Hamad Medical Corporation, Doha, Qatar

**Keywords:** aortic valve regurgitation, fixed orthodontic appliance, infective endocarditis, *Streptococcus sanguinis*

## Abstract

**Objectives:**

The risk of endocarditis is caused by Streptococcus viridans, which is found in the mouth and can cause dental issues. Men over 45 are at higher risk of endocarditis, and orthodontic appliances can disrupt mouth bacteria, increasing the risk of endocarditis and oral streptococci. Good oral hygiene and checkups can prevent complications, and patients in orthodontic treatment should be monitored for heart conditions and educated on oral hygiene.

**Case presentation:**

A 31-year-old male with known aortic valve disease was admitted for infective endocarditis and was treated with intravenous ceftriaxone.

**Conclusions:**

Fixed orthodontic appliances increase the risk of endocarditis and oral Streptococci, but patients can reduce these risks by maintaining oral hygiene and adhering to home oral hygiene routines; regular professional dental cleanings can also help maintain oral health during fixed orthodontics.

## Introduction

Braces can cause infections and complications during the adjustment phase, as they provide a breeding ground for bacteria. To minimize the risk, regular dental check-ups, good oral hygiene, and antibiotics in high-risk scenarios are necessary [1]. Orthodontic treatment can cause infective endocarditis in patients with heart conditions or weakened immune systems due to bacteria in the mouth. Dental procedures can also lead to this infection caused by other bacteria. Although the risk of *Streptococcus sanguinis* causing endocarditis is low, it can still be a concern based on different factors [2]. Patients with pre-existing medical conditions have a higher risk of infection after orthodontic treatment [3]. The American Heart Association recommends antibiotic prophylaxis to prevent infective endocarditis before dental procedures. Dental professionals must follow strict infection control measures to reduce infection risks [3]. The risk of infective endocarditis after orthodontic procedures is low, estimated at less than one in 100,000 cases. *Streptococcus* species are the most common microorganisms responsible for these infections [4].

## Case presentation

A 31-year-old male patient, from Pakistan, known to have had aortic valve disease since childhood, which is moderately to severely aortic valve disease, and orthodontic braces were in place, was admitted to the hospital 6 weeks before for fever and headache, and was suspected to have infective endocarditis. Thus, he was treated accordingly and was discharged.

After his discharge on the previous admission, he was well; he did not have any episodes of fever for about a week, then he began to start to have a fever again, which had no particular time, not associated with cough, chest pain, or shortness of breath. The patient sleeps with one below his head and has no history of Paroxysmal nocturnal dyspnea (PND). He claims that he participates in diving activities without any incidents. He is a non-smoker.

So, going back 6 weeks from this admission, he presented to the hospital with a fever that was remittent in type and chills, associated with a headache for about 18 days before that, with no evidence of bacteremia, vegetation or masses by the transthoracic echocardiography. He was given broad-spectrum antibiotics and was discharged from the hospital.

On the recent admission, he reported that one week from discharge, he continued to have persistent fever, chills, and a generalized headache. He denied any chest pain or shortness of breath, palpitations, or orthopnea on room air. At his presentation to the emergency department (EMD), the patient was febrile at around 38.4 °C with a saturation of 98 % on room air, a heart rate of 112 beats per minute, and a blood pressure of 139 over 76 mmHg. Despite his vital signs, he appears to be calm. His physical examination of the cardiovascular system revealed possible increased redness in the nail beds in his hands, but no clear-cut splinter hemorrhage. There is no pedal edema. JVP was not raised. Precordium examination showed a long diastolic murmur radiating to the neck with slightly muffled S^2^ sounds in keeping with his known aortic regurgitation. Additionally, the respiratory examination was essentially unremarkable except for a very faint few crepitations in the right base of doubtful significance, and there was no hepatic-splenomegaly on the abdominal side.

Laboratory tests were significant for leukocytosis (Table 1). Two Blood cultures were drawn from the patient’s arm using an aseptic technique and inoculated onto culture media. The blood cultures showed the growth of small alpha-hemolytic colonies on the agar plate, identified as *S. sanguinis* by Gram staining and biochemical tests, which was sensitive to ceftriaxone, penicillin, and vancomycin on the antimicrobial susceptibility testing (AST) (Table 2). An infectious disease specialist saw the patient and recommended starting intravenous ceftriaxone 2 g daily and performing transthoracic echocardiography (TTE).

**Table 1:** Laboratory results.

Laboratory tests	Values	Normal values
WBC	15	4–10 × 10^3^/µL
RBC	4.8	4.5–5.5 × 10^6^/µL
Hb	12.0	13–17 gm/dL
Hct	37.0	40–50 %
MCV	76.8	83–101 fL
MCH	24.9	27–32 pg
MCHC	32.4	31.5–34.5 gm/dL
Platelet	320	150–410 × 10^3^/µL
Neutrophils, %	74.4	
Lymphocytes, %	18.9	
Monocytes, %	6.1	
Eosinophils, %	0.1	
Basophils, %	0.5	
Urea	3.9	2.5–7.8 mmol/L
Creatinine	62	62–106 µmol/L
Sodium	134	133–146 mmol/L
Potassium	4.5	3.5–5.3 mmol/L
Chloride	96	95–108 mmol/L
Bicarbonate	29	22–29 mmol/L
Magnesium	0.82	0.70–1 mmol/L
Bilirubin total	11	0–21 µmol/L
Total protein	84	60–80 gm/L
Albumin	31	35–50 gm/L
Alkaline phosphatase	77	40–29 U/L
Alanine transaminase, ALT	17	0–41 U/L
Aspartate aminotransferase, AST	22	0–40 U/L
C-reactive protein, CRP	91.8	0–5 mg/L
Iron	5	6–35 µmol/L
TIBC	35	45–80 µmol/L
Fe % saturation	14	15–45
Transferrin	1.4	2–3.6 gm/L
Ferritin	536.0	48–420 µg/L
HbA1C	5.7 %	<5.7–6.4 %
SARS-CoV-2 PCR	Negative	Negative

WBC, white blood cell; RBC, red blood cell; Hb, hemoglobin; Hct, hematocrit; MCV, mean corpuscular volume; MCH, mean corpuscular hemoglobin; MCHC, mean corpuscular hemoglobin concentration; TIBC, total iron-binding capacity; SARS-CoV-2, severe acute respiratory syndrome coronavirus 2; PCR, polymerase chain reaction.

**Table 2:** Antimicrobial susceptibility test (AST).

Antimicrobial agent	*Streptococcus sanguinis* (*Streptococcus viridans* group)
Ceftriaxone	Sensitive
Penicillin	Intermediate
Vancomycin	Sensitive

**Figure 1:**
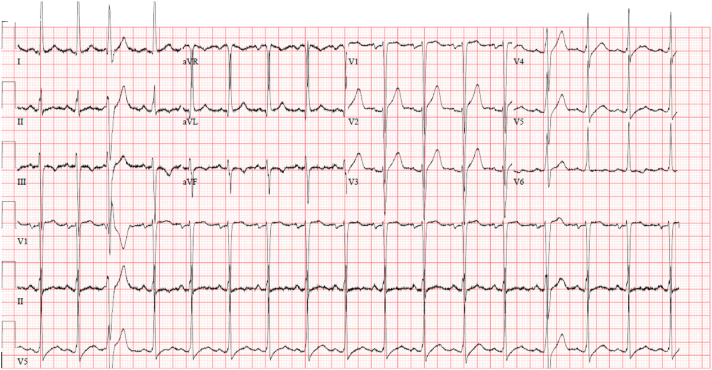
Electrocardiogram (ECG) showed Sinus Tachycardia with occasional ventricular premature complexes possible left atrial enlargement and ventricular hypertrophy with no specific ST & T-wave abnormality.

**Figure 2:**
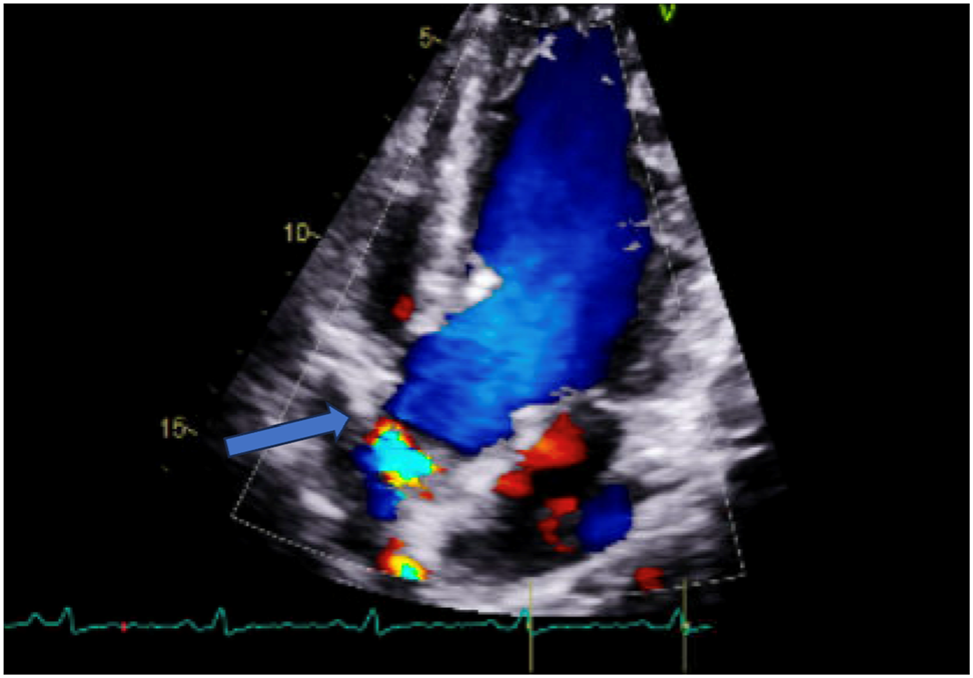
Transthoracic echocardiogram (TTE); revealed moderate aortic valve regurgitation.

**Figure 3:**
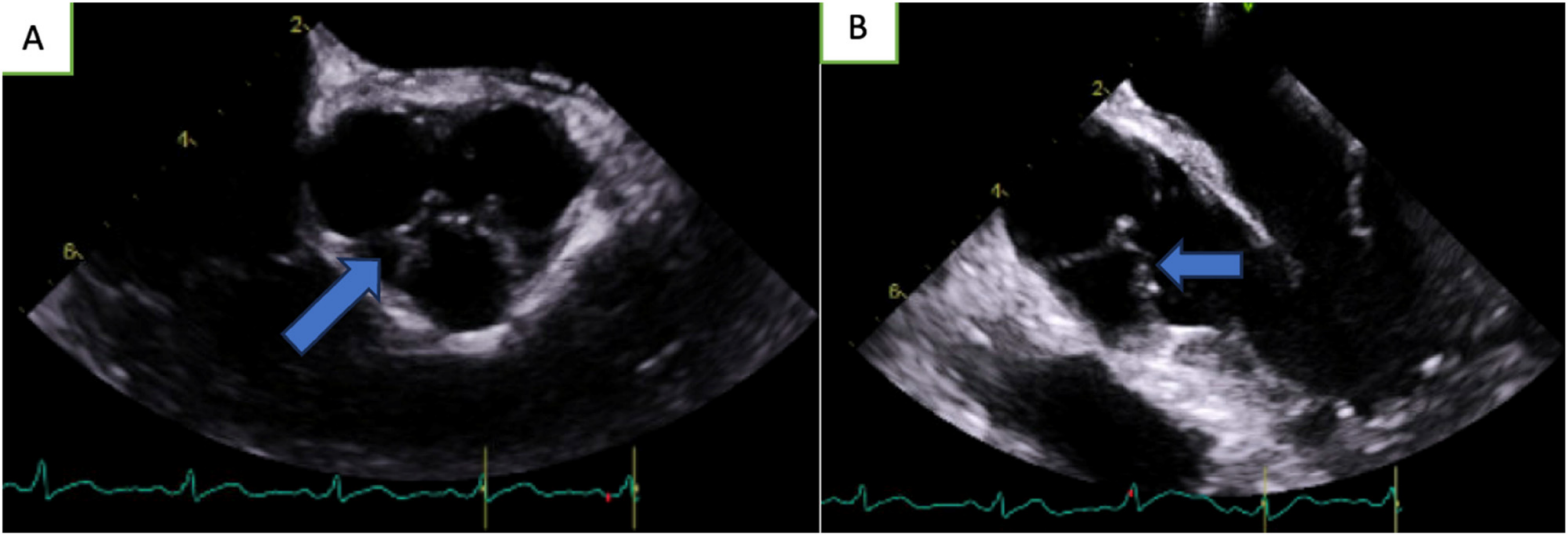
Transesophageal echocardiogram (TEE); (A and B) revealed no masses or vegetation seen in this study, there is no subaortic membrane, but there is a false tendon in the left ventricle (LV) close to the left ventricular outflow tract (LVOT) without hemodynamic effect.

**Figure 4:**
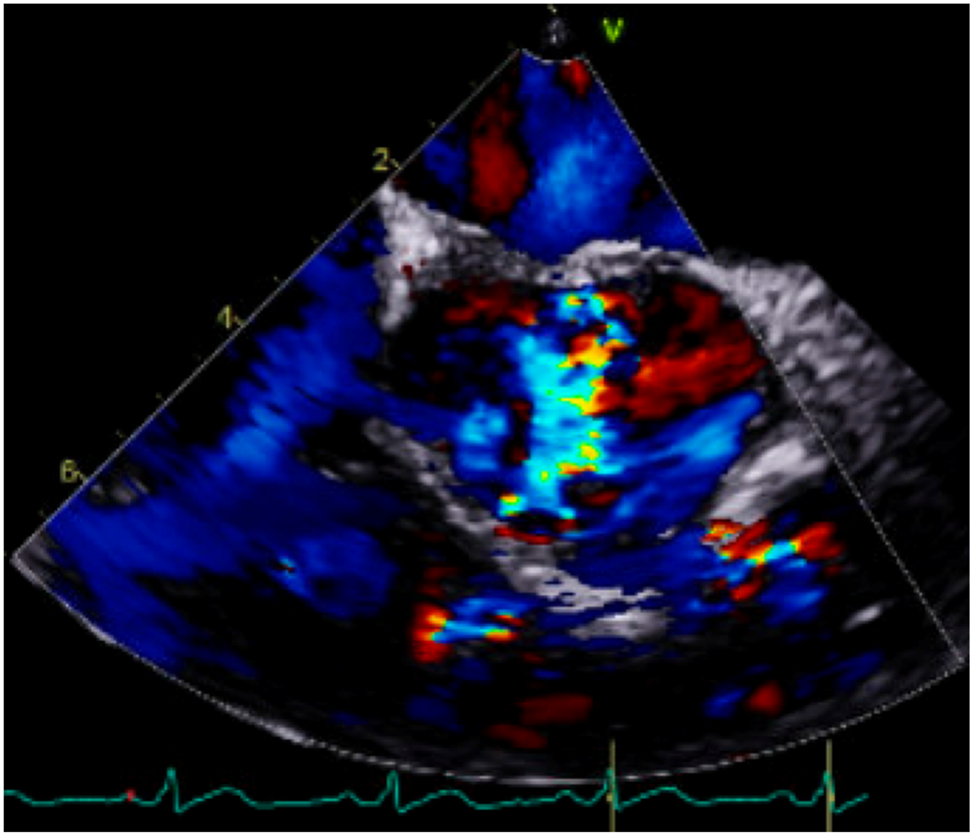
Transesophageal echocardiogram (TEE); moderate to severe aortic regurgitation.

His electrocardiogram (ECG) showed the features that go with aortic regurgitation disease: left atrial enlargement and ventricular hypertrophy (Figure 1). Afterwards, we went advance in his investigations through a transthoracic echocardiogram (TTE) revealed a left ventricle (LV) ejection fraction of 57 %, normal global systolic function, moderate aortic valve regurgitation, highly possible subaortic membrane with no significant systolic gradient across the Left ventricular outflow tract (LVOT), and no regional wall motion abnormality (Figure 2). Then, the patient was recommended to go under a transesophageal echocardiogram (TEE) to investigate the condition further, confirming that there are no masses or vegetation seen in this study, there is no subaortic membrane, but there is a false tendon in the left ventricle (LV) close to the Left ventricular outflow tract (LVOT) without hemodynamic effect, and evidence of demonstration of moderate to severe aortic regurgitation (Figures 3 and 4).

The hospital medicine, infectious disease, and cardiology team evaluated the patient daily. His blood cultures showed *S. sanguinis* by Gram staining and biochemical tests during his hospital stay. Additionally, this case is unique because there are signs of infective endocarditis with no evidence of vegetation or masses on either the TTE or TEE. The patient was advised to receive a course of intravenous ceftriaxone 2 g daily for six weeks, and then will have to follow up in the General medicine and Cardiology clinics with a couple of planned echocardiograms and cardiac imaging to assess the heart anatomy.

### Research ethics

This patient provided oral and signed written consent for the use of his clinical materials in the principles of the institutional ethical standards, and the national research committee conducted the study ethical standards and the national research committee.

### Informed consent

Written informed consent was obtained from the patient to publish this case report and any accompanying images.

## Discussion

*Streptococcus viridans* is a group of alpha-hemolytic streptococci that are found in the mouth’s normal flora. They are usually responsible for dental caries (*Streptococcus mutans*, *S. sanguinis*), pericoronitis, and subacute infective endocarditis. Although *S. sanguinis* endocarditis is a relatively rare condition, it is typically more common in older individuals with pre-existing heart conditions or intravenous drug use. In this case, a 31-year-old male was diagnosed with this infection after orthodontic bracing. Dental procedures have been recognized as potential sources of endocarditis, but the specific association with *S. sanguinis* is less commonly reported in the literature [5], 6].

According to the Duke criteria, the diagnosis of infective endocarditis was supported by one major criterion that was met, two positive blood cultures with *S. sanguinis*, and two minor criteria: temperature of 38.4 °C and the presence of Janeway lesions. The use of antibiotic therapy with Ceftriaxone with the extended course will help to clear the bacteria from the blood [6]. After evaluating the potential risk of penicillin tolerance or resistance, it was decided with confidence to forgo the use of penicillin. Up to 13 % of strains have exhibited such resistance, and therefore, an alternative treatment was chosen [7].

The patient’s endocardium infection was successfully treated with a six-week monotherapy of intravenous ceftriaxone – an antibiotic that has been proven to be highly effective in treating infective endocarditis and is recommended as the first-line agent by the guidelines of the Infectious Diseases Society of America (IDSA). In the case of infective endocarditis caused by *S. sanguinis*, the duration of treatment is typically 4–6 weeks, and it can be adjusted depending on the patient’s response to treatment. The choice of antibiotic therapy was based on the susceptibility profile of the organism and the patient’s clinical condition, ensuring the best possible outcome. Although infective endocarditis caused by *S. sanguinis* has a low mortality rate, administering prompt and appropriate treatment is crucial to avoid complications and ensure positive outcomes. It is imperative to note that developing complications may lead to a more complicated clinical course and worse outcomes. However, with timely and adequate treatment, the prognosis for this condition is generally favorable [8].

The guidelines provided by the American Heart Association (AHA) and the European Society of Cardiology (ESC) are an indispensable resource for preventing infective endocarditis in high-risk patients undergoing dental procedures, including orthodontic treatments. The 2007 AHA guidelines are crystal clear and provide comprehensive instructions regarding the use of antibiotic prophylaxis for patients with medical conditions such as prosthetic cardiac valve, previous infective endocarditis, and congenital heart disease (CHD), among others. For patients with these conditions undergoing orthodontic procedures, the AHA strongly recommends antibiotic prophylaxis with amoxicillin or clindamycin, except in cases of penicillin allergy. The ESC guidelines are in line with the AHA guidelines and also recommend antibiotic prophylaxis with amoxicillin or clindamycin for patients with specific conditions, including prosthetic heart valves and congenital heart disease, among others. These guidelines are based on extensive research and are widely accepted and followed by medical professionals worldwide, which makes them the gold standard for preventing infective endocarditis in high-risk patients undergoing dental procedures [9].

Purpura fulminans is an extremely rare manifestation of pneumococcal bacteremia in immunocompetent individuals, highlighting the critical importance of pneumococcal vaccination. This case emphasizes prevention, early recognition, and evidence-based management to mitigate the risks of invasive pneumococcal disease [10].

## Conclusions

It is worth considering the unique association between endocarditis and oral streptococci caused by fixed orthodontic appliances. What sets this apart is the fact that the patients affected are usually young adults, between the ages of 20–35 years – which is not typically seen in other cases of endocarditis.

After the introduction of orthodontic fixed appliances in the oral cavity, there is a definite change in the bacterial flora, which leads to an increased concentration of acidogenic bacteria. The subgingival bacterial flora multiplies during fixed orthodontics and the bonding materials used in the appliances retain biofilms. However, a coordinated effort between the orthodontist and dental hygienist can significantly reduce oral health risks associated with orthodontic treatment. Educating and motivating the patient to observe oral hygiene and adhere to the home oral hygiene routine is imperative. Regular mechanical tooth cleaning by a professional dental hygienist can help maintain good oral hygiene during fixed orthodontics and considerably reduce oral health risks.
